# Occupational Fitness Assessments in Healthcare Workers with Psychiatric Disorder

**DOI:** 10.1192/j.eurpsy.2025.1812

**Published:** 2025-08-26

**Authors:** C. Sridi, F. Chelly, A. Fki, N. Belhaj, S. Ksibi

**Affiliations:** 1 Occupational Medicine Department, Sahloul University Hospital; 2 Occupational Medicine Department, Faculty of Medicine of Sousse, Sousse, Tunisia

## Abstract

**Introduction:**

Healthcare professionals are exposed to numerous stressors in their work environment, which can lead to psychiatric disorders.

**Objectives:**

To describe the profile of healthcare professionals who requested a fitness-for-duty evaluation due to a psychiatric disorder and to identify the recommended workplace adaptations.

**Methods:**

A retrospective study was conducted in the University Hospital Sahloul of Sousse (Tunisia) over a three-year period (2022-2024). Data from healthcare staff who consulted for fitness-for-duty evaluations related to psychiatric conditions were collected, including age, work experience, job position, psychiatric diagnosis, and fitness-for-duty decisions.

**Results:**

The study included 13 healthcare professionals comprising 6 senior technicians, 6 nurses, and 1 worker. The average age was 45 ± 3.2 years, with an average seniority of 11 ± 2.8 years. The most common psychiatric conditions were anxiety-depressive syndrome (10 cases), post-traumatic stress disorder (2 cases), and obsessive-compulsive disorder (1 case). All patients were on antidepressants and/or anxiolytics at the time of consultation. The fitness-for-duty decisions included recommendations for avoiding night shifts and exposure to stressful situations.

**Conclusions:**

Psychiatric disorders among healthcare workers present significant challenges for occupational health services. Workplace adaptations, such as avoiding night shifts and stressful environments, may be necessary to facilitate a return to or continuation of work.

**Disclosure of Interest:**

None Declared

